# OpenMinds on Mental Health Literacy: A Reflective Journey of a Medical Student

**DOI:** 10.1192/bjo.2022.151

**Published:** 2022-06-20

**Authors:** Jashan Selvakumar, Jiann Lin Loo, May Honey Ohn

**Affiliations:** 1 St George's University of London, London, United Kingdom; 2 Betsi Cadwaladr University Health Board, Wrexham, United Kingdom; 3 Croydon University Hospital, London, United Kingdom

## Abstract

**Aims:**

As a medical student from a local university, the first author undertook a mental health education course, i.e. OpenMinds at the King's College University. The aim of the course is to improve literacy about key mental health issues that children and adolescents face and the stigma against mental illnesses. Upon completion of training, a medical student will be able to lead intervention workshops to share the mental health knowledge with local school audiences on these issues, promote early detection of mental illnesses among the audiences and their peers with the aim of improving health-seeking behaviour by providing information of where to access help to reduce the duration of untreated illness. This article is aimed to describe the personal reflective experience of a medical student and the lessons learnt.

**Methods:**

The OpenMinds course was an eight-week workshop on important mental health topics such as depression, anxiety, coping strategies and psychosis. This was followed by a session on effective teaching detailing various techniques including maintaining children's concentration, increasing engagement by utilising different learning techniques, safeguarding and maintaining well-being during conversations about difficult and sensitive topics.

**Results:**

After attending the OpenMinds educational workshop, the first author had delivered three workshops (one primary school and two secondary schools) as part of the bigger organising team from the other university. Overall, the verbal feedback from the local schools on the workshops was positive (Kirkpatrick's evaluation outcome level one). The challenge faced was virtual teaching due to the COVID-19 pandemic which meant not being able to read facial expressions or body language while delivering information. This limitation could be mitigated by having a trained teacher moderating the sessions on-site and making sure the workshops ran smoothly. Online lessons emphasised the use of technology which was proven to be useful as videos and other audiovisual aids had the ability to keep the children engaged and provide different sources of learning concurrently.

**Conclusion:**

Having participated in this course, the first author has learned teaching skills and a better way of communicating mental health issues to vulnerable audiences. Although face-to-face workshops are still not possible at the time of writing, the first author is keen to set up an OpenMinds branch at his university and be able to share with his fellow colleagues these skills in the future.

